# Assessing Awareness, Knowledge, Perceptions, and Safety Practices Among Patients Regarding MRI Scans in the Qassim Region, Saudi Arabia

**DOI:** 10.7759/cureus.98240

**Published:** 2025-12-01

**Authors:** Ziyad A Almushayti, Reef A Alsuhaibani, Razan S Alharbi, Joud M Almotairi, Areen E Almatham, Wamidh M Alkhalifah, Sali F Alharbi

**Affiliations:** 1 Department of Radiology, Qassim University, Buraydah, SAU; 2 College of Medicine, Qassim University, Buraydah, SAU

**Keywords:** awareness, knowledge, magnetic resonance imaging (mri), radiological modalities, safety

## Abstract

Introduction: Magnetic resonance imaging (MRI) is a non-invasive radiological technique that generates detailed three-dimensional images of the body. While it offers advanced diagnostic capabilities, MRI poses potential risks, such as interactions with static magnetic fields and radiofrequency exposure, particularly when safety guidelines are not properly followed or outdated information is used.

Methods: The study utilized a cross-sectional research design to assess patients' awareness, knowledge, perceptions, and safety regarding MRI scans in the Qassim region. A total of 412 participants were involved, and data were collected online through social media platforms such as WhatsApp, Twitter, and Telegram. The data was then analyzed using IBM SPSS Statistics for Windows, Version 27 (Released 2019; IBM Corp., Armonk, New York, United States) to gain key insights.

Results: More than half of the participants (238, 57.8%) believed they were knowledgeable about MRI risks and safety requirements. The study further revealed that less than half (181, 43.9%) of the participants had received information about MRI risks and the necessary safety precautions for the examination, while 231 (56.1%) had not. Among those who received information, the most common source was the internet (100, 55.2%). Additionally, most participants were informed that clothes (289, 70.1%), paper (237, 57.5%), and plastic buttons (240, 58.3%) are safe items during MRI examinations. The socio-demographic variables, namely gender, age, education level, and employment status, did not show a statistically significant difference in relation to knowledge about MRI (p-value > 0.05).

Conclusion: The study found that while over half of participants felt informed about MRI risks and safety, there are still significant knowledge gaps. Most who had undergone an MRI felt their experiences increased their understanding. Demographic factors did not significantly affect knowledge levels. The findings suggest the need for targeted educational interventions, with online platforms and social media being effective tools for reaching a broader audience.

## Introduction

Magnetic resonance imaging (MRI) is a radiological technique that produces three-dimensional (3D) images of the body. It is among the most widely used non-invasive methods, providing advanced anatomical, physiological, and diagnostic information [1-5]. Since its development in the late 1970s, MRI technology has significantly advanced, making it indispensable in modern medicine [6]. However, the increased use of strong radiofrequency transmission coils and powerful static magnetic fields (SMFs) makes safety precautions more critical than ever [7].

While MRI avoids the use of ionizing radiation, a key advantage over other imaging methods, it still poses risks. These include potential interactions between SMFs and the human body, interference with medical implants, and exposure to radiofrequency fields [8]. Additionally, allergic reactions to contrast agents may occur [8].

Although widespread use of MRI, many patients lack a comprehensive understanding of MRI safety protocols. This gap of knowledge can lead to anxiety, difficulty remaining still during the scan, and ultimately, poor image quality due to motion artifacts [1]. Research shows that many MRI-related injuries, and even rare fatalities, result from failure to follow safety guidelines or reliance on outdated information about implants [9]. Patient education about MRI safety is essential for reducing risks and improving scan quality [1,2].

Given these safety concerns, assessing patients' awareness, knowledge, and perceptions of MRI safety is essential. This study evaluates these factors in the Qassim Region, Saudi Arabia, where cultural and healthcare dynamics may influence patient understanding. Although MRI safety has been widely studied, few investigations have focused on this specific region, making this research critical for identifying knowledge gaps and enhancing patient safety.

Furthermore, a lack of patient knowledge can lead to inefficiencies in MRI workflow, lower compliance, and increased scan times. By addressing common misconceptions and improving patient education, healthcare providers can reduce these risks [10,11]. Effective communication strategies, such as educational booklets, videos, and personalized consultations, have proven to enhance patient understanding and reduce anxiety [12]. This research aims to contribute to these efforts by offering insights into how patient awareness can be improved, ensuring safer and more efficient MRI procedures.

Although awareness and safety-oriented knowledge have been studied across multiple areas of Saudi Arabia, few studies have been held in the Qassim region with various cultural factors, differing degrees of health literacy, and different pathways to healthcare that affect perceptions. The purpose of this study was to assess the awareness, knowledge, perceptions, and safety attitudes towards MRI in the general population in Qassim. To focus the study further, we described the general levels of awareness and perception and whether demographic characteristics were associated with MRI knowledge. These findings will help healthcare providers practice better education and enhance patient safety and compliance in MRI scans.

## Materials and methods

This descriptive cross-sectional study aimed to assess patients' awareness, knowledge, perceptions, and safety concerns regarding MRI scans in the Qassim region of Saudi Arabia. The study was conducted between July 2024 and August 2024. The inclusion criteria were as follows: adults of both genders aged 18 years or older, living in the Qassim region. Individuals younger than 18 years and not residents of the Qassim region were excluded from the study.

Participants were recruited using convenience sampling through social media platforms, including X (Twitter), WhatsApp, and Telegram-common communication channels in Qassim. Because online recruitment can disproportionately attract younger and more educated individuals, we attempted to distribute the survey across diverse community groups to reduce sampling bias. However, the potential for overrepresentation remains a noted limitation. The survey was adapted from a study by Alahmari et al. [13], which focused on MRI safety awareness after seeking permission. The questionnaire comprised four sections: demographic information, knowledge of MRI safety and potential risks, preferred sources of information about MRI, and prior experiences with MRI procedures. An Arabic-language, self-administered questionnaire was distributed to participants. The knowledge section consisted of items formatted using a three-point response scale (“Agree,” “Disagree,” and “Unsure”). Each item had one correct answer based on established MRI safety guidelines. For scoring, correct responses were assigned 1 point, while incorrect and “Unsure” responses were assigned 0 points.

The ethical approval was obtained from the Regional Research Ethics Committee in the Qassim region (approval number 607/46/424) dated July 17, 2024. Informed consent was obtained from all participants. They were informed that participation was voluntary, that their responses would remain confidential, and that they could withdraw from the study at any time. The study’s objectives, as well as any potential risks and benefits, were communicated clearly before obtaining consent.

The required sample size of 385 participants was calculated using the Raosoft online calculator (Raosoft Inc., Seattle, WA, US), based on the Qassim region’s population of 1,488,285, as reported in a 2019 survey by the General Authority for Statistics in Saudi Arabia. Prior to data collection, a pilot test of 15 participants was conducted to assess the clarity and acceptability of the adapted items. Based on reviewer recommendations, internal reliability (Cronbach’s α) was reassessed for the adapted version used in this study and yielded a value of α = 0.74, indicating acceptable internal consistency.

Data collected from participants were initially entered into Microsoft Excel (Microsoft Corp., Redmond, WA, USA) for preliminary cleaning, validation, and organization. Afterward, the dataset was transferred to IBM SPSS Statistics for Windows, Version 27 (Released 2019; IBM Corp., Armonk, New York, United States) for comprehensive statistical analysis. Descriptive statistics, including frequencies and percentages, were used to summarize data. These results were visually represented using tables and charts. To examine potential associations between participants' socio-demographic variables and their level of knowledge about MRI surgery, inferential statistics were applied using the chi-square test. A p-value of less than 0.05 was considered statistically significant throughout the analysis.

## Results

The study included a total of 412 participants from the Qassim region. The majority were female, 299 (72.6%), with the remaining 113 (27.4%) being male. Age distribution showed that over half (269, 65.3%) of the participants were between 18 and 25 years old, while 88 (21.4%) were aged 26 to 39 years, and 55 (13.3%) were over 40 years old. Regarding education, the majority held a Bachelor's degree (231, 56.1%), followed by high school graduates (113, 27.4%), diploma holders (37, 9.0%), and postgraduates (31, 7.5%). In terms of employment status, more than half (233, 56.6%) were students, 104 (25.2%) were employed, and 75 (18.2%) were unemployed (Table 1).

**Table 1:** Demographic information. Social demographic variables are presented as frequencies (n) and proportions (%). The questionnaire was adapted from Alahmari et al. [13].

Demographic information	Category	Frequency and proportion, n (%)
Gender	Male	113 (27.4)
	Female	299 (72.6)
Age	18-25 years	269 (65.3)
	26-39 years	88 (21.4)
	More than 40 years	55 (13.3)
Education level	Bachelor’s degree	231 (56.1)
	Diploma	37 (9.0)
	High school	113 (27.4)
	Postgraduate	31 (7.5)
Employment status	Employee	104 (25.2)
	Unemployed	75 (18.2)
	Student	233 (56.6)

Table 2 shows that more than half of the participants, 238 (57.8%), believe they have knowledge about MRI risks and safety needs. Nearly half of them, 193 (46.8%), were aware that some objects inside the body may be incompatible with an MRI exam. The results also reveal that most participants would not be comfortable having an MRI if they have worked with metal filings, 218 (52.9%), and they understand that metallic objects inside the body can be twisted by the magnetic field and cause serious injuries, 180 (43.7%). A significant proportion was unsure whether MRI noise had an effect on cochlear function (174, 42.2%), and that MRI can slow blood flow and increase blood pressure (231, 56.1%). Moreover, the majority agreed that magnetic and radio frequency fields have side effects (234, 56.8%), and others were unsure whether the MRI machine can alter the heart's beating rhythm (220, 53.4%). Most participants were unsure that the levels of magnetism used in an MRI unit can heat the body’s tissues (243, 59.0%), while others believed that an MRI exam was unsafe to have during pregnancy (242, 58.7%). Finally, the majority, 229 (55.6%), were unsure that all people with tattoos cannot have an MRI examination.

**Table 2:** Knowledge about the MRI procedure and its safety. Data presented as frequencies (n) and proportions (%). The questionnaire was adapted from Alahmari et al. [13].

Knowledge regarding MRI	Agree	Disagree	Unsure
I believe I have knowledge about MRI risks and safety needs.	238 (57.8%)	93 (22.6%)	81 (19.7%)
Some objects which may have been placed inside of my body may be incompatible with my MRI exam.	193 (46.8%)	85 (20.6%)	134 (32.5%)
I am OK to have an MRI examination if I have worked with metal filings.	102 (24.8%)	218 (52.9%)	92 (22.3%)
Metallic objects inside the body cannot be twisted by the magnetic field and cause serious injuries.	78 (18.9%)	180 (43.7%)	154 (37.4%)
There is no effect of MRI noise on cochlear function.	96 (23.3%)	142 (34.5%)	174 (42.2%)
MRI used in hospitals for diagnosis can slow your blood flow and cause increased blood pressure?	82 (19.9%)	99 (24.0%)	231 (56.1%)
Magnetic and radio frequency fields have side effects.	234 (56.8%)	61 (14.8%)	117 (28.4%)
An MRI machine can alter the hearts beating rhythm?	104 (25.2%)	88 (21.4%)	220 (53.4%)
The levels of magnetism used in an MRI unit cannot heat your bodies tissues.	92 (22.3%)	77 (18.7%)	243 (59.0%)
An MRI exam is safe to have during pregnancy (for female participant).	55 (13.3%)	242 (58.7%)	115 (27.9%)
All people with tattoos cannot have an MRI examination.	60 (14.6%)	123 (29.9%)	229 (55.6%)

Table 3 shows that most of the participants deemed clothing (289, 70.1%), paper (237, 57.5%), and plastic buttons (240, 58.3%) as safe, with pillows also being seen as safe by a majority (269, 65.3%). Items like food and water had mixed perceptions, with 183 (44.4%) and 191 (37.3%) of participants considering them safe, respectively. Conversely, items such as coins (339, 82.3%), watches (334, 81.1%), and rings (323, 78.4%) were predominantly viewed as unsafe. Eyeglasses with metallic frames (319, 77.4%) and mobile phones (318, 77.2%) were also widely considered unsafe. Furthermore, nearly two-thirds of the participants considered ferromagnetic clips (278, 67.5%), pens (272, 66.0%), baby carts (261, 63.3%), and wheelchairs (253, 61.4%) to be unsafe during an MRI. These findings reveal a diverse range of perceptions regarding safe and unsafe items during MRI examinations, highlighting the need for increased awareness and accurate information to improve understanding.

**Table 3:** Safe/unsafe items in MRI examination. Data presented as frequencies (n) and proportions (%). The questionnaire was adapted from Alahmari et al. [13].

Item	Safe	Unsafe	Not sure
Rings	31 (7.5%)	323 (78.4%)	58 (14.1%)
Food	183 (44.4%)	100 (24.3%)	129 (31.3%)
Medication	143 (34.7%)	108 (26.2%)	161 (39.1%)
Eyeglasses with metallic frames	28 (6.8%)	319 (77.4%)	65 (15.8%)
Watches	22 (5.3%)	334 (81.1%)	56 (13.6%)
Coins	18 (4.4%)	339 (82.3%)	55 (13.3%)
Clothing	289 (70.1%)	48 (11.7%)	75 (18.2%)
Credit cards	61 (14.8%)	206 (50.0%)	145 (35.2%)
Paper	237 (57.5%)	54 (13.1%)	121 (29.4%)
Contact lenses	63 (15.3%)	191 (46.4%)	158 (30.9%)
Water	191 (37.3%)	63 (12.3%)	158 (38.3%)
Cardiac devices (e.g. pacemaker)	50 (12.1%)	174 (42.2%)	188 (45.6%)
Ferromagnetic clips	28 (6.8%)	278 (67.5%)	106 (25.7%)
Perfume	174 (42.2%)	72 (17.5%)	166 (40.3%)
Metal filings	46 (11.2%)	231 (56.1%)	135 (32.8%)
Plastic buttons	240 (58.3%)	63 (15.3%)	109 (26.5%)
Wheelchair	37 (9.0%)	253 (61.4%)	122 (29.6%)
Baby cart	32 (7.8%)	261 (63.3%)	119 (28.9%)
Mobile	16 (3.9%)	318 (77.2%)	78 (18.9%)
Pillow	269 (65.3%)	34 (8.3%)	109 (26.5%)
Pens (plastic or metallic)	27 (6.6%)	272 (66.0%)	113 (27.4%)

**Figure 1:**
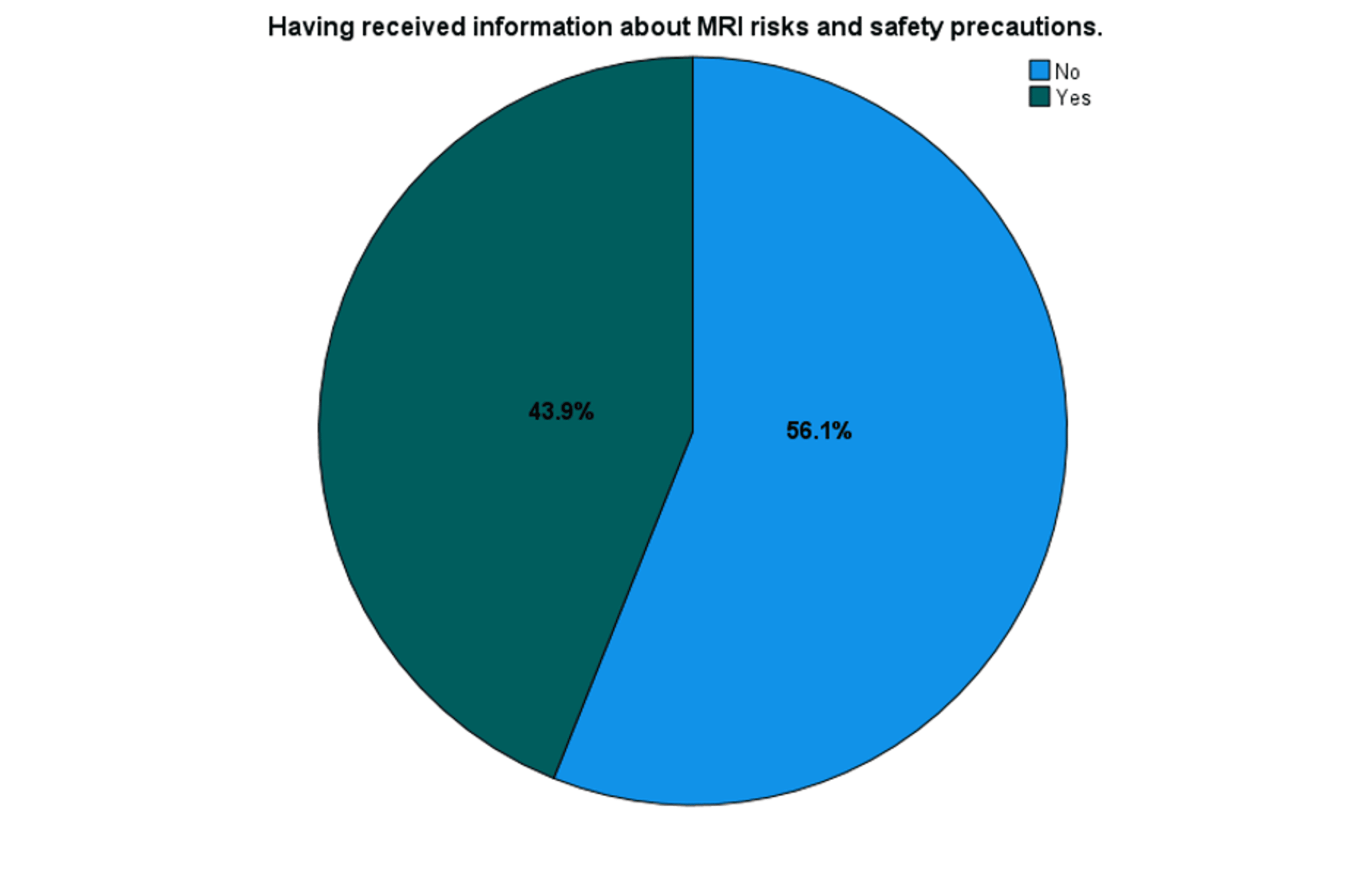
Distribution of participants who received information regarding MRI risks and safety precautions.

Figure 1 presents the distribution of participants who received information about MRI risks and the safety precautions required for the examination. It shows that 181 participants (43.9%) had received the information, while more than half, 231 participants (56.1%), had not.

**Figure 2:**
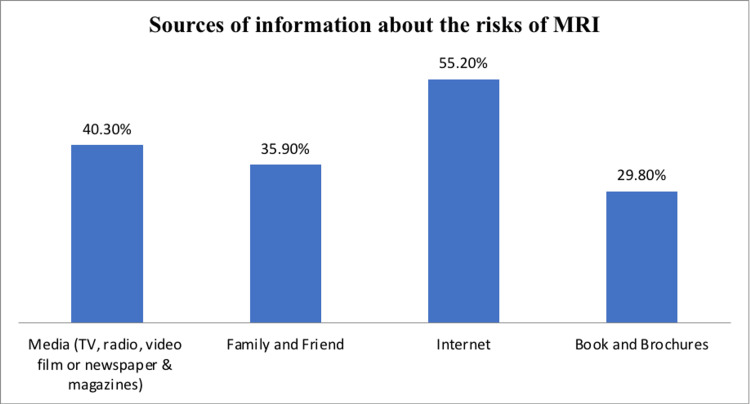
Sources of information about the risks of MRI.

Figure 2 illustrates the sources of information about MRI risks and the required safety precautions. The most common source was the internet (100 participants, 55.2%), followed by media sources such as TV, radio, videos, and newspapers or magazines (73 participants, 40.3%), family and friends (65 participants, 35.9%), and books or brochures (54 participants, 29.8%).

Table 4 shows that below half (181, 43.9%) of participants received information about MRI risks and safety precautions required for the examination, while 231 (56.1%) did not. When asked about their preferred sources for obtaining knowledge about MRI risks and safety, the majority favored face-to-face communication (175, 42.5%), followed by the internet (127, 30.8%), books and brochures (66, 16.0%), media (29, 7.0%), and family and friends (15, 3.6%). These findings highlight the importance of providing accurate information to promote safety and proper use of MRI, as well as the growing role of the internet in disseminating reliable health information.

**Table 4:** Sources of information about MRI. Data presented as frequencies (n) and proportions (%). The questionnaire was adapted from Alahmari et al. [13].

MRI sources of information	Category	n (%)
Have received information about MRI risks and the safety precautions required for an examination?	Yes	181 (43.9)
	No	231 (56.1)
Have you ever received information about the risks of MRI and the safety precautions required for the examination? N=181	Media (TV, radio, video film, or newspapers and magazines)	73 (40.3)
	Family and friends	65 (35.9)
	Internet	100 (55.2)
	Book and brochures	54 (29.8)
Which sources you prefer to get your knowledge about MRI risks and safety?	Media (TV, radio, video film, or newspapers and magazines)	29 (7.0)
	Family and friends	15 (3.6)
	Internet	127 (30.8)
	Book and brochures	66 (16.0)
	Face to face	175 (42.5)

Table 5 shows that slightly more than a third (143, 34.7%) of the respondents had been examined by MRI before, while 269 (65.3%) of the participants had never been. Among the 143 participants who had undergone an MRI, more than half, 85 (59.4%), had only one exam, 24 (16.8%) had two exams, 11 (7.7%) had three exams, and 23 (16.1%) had more than three exams. In terms of their experience with MRI safety and precautions, 93 (65.0%) found their actual experience to be similar to their expectations, while 50 (35.0%) found it to be very different. Since their first MRI exam, 83 (58.0%) reported an increase in knowledge about MRI safety and risks, 18.2% did not experience an increase, and 34 (23.8%) were unsure. When asked about their knowledge of MRI safety, including precautions and risks after their previous exam, nearly three-quarters of the participants (106, 74.1%) expressed satisfaction. This highlights the growing desire and interest in gaining knowledge among the majority of participants.

**Table 5:** MRI examination experience and knowledge assessment. Data presented as frequencies (n) and proportions (%). The questionnaire was adapted from Alahmari et al. [13].

MRI sources of information	Category	Frequency and proportion, n (%)
Have ever been examined by MRI before?	Yes	143 (34.7)
	No	269 (65.3)
How many times you have examined by MRI machine? N=143	One	85 (59.4)
	Two times	24 (16.8)
	Three times	11 (7.7)
	>Three times	23 (16.1)
How did your actual experience of MRI safety and the precautions required (before and during) your examination, compare with what you expected? N=143	My actual experience was similar to what I had expected.	93 (65.0)
	My actual experience was very different from what I had expected.	50 (35.0)
Has your knowledge about MRI safety and risks increased since the first MRI exam? N=143	Yes	83 (58.0)
	No	26 (18.2)
	Not sure	34 (23.8)
How would you evaluate your knowledge about MRI safety, that is, the precautions and risks of the examination after the previous exam? N=143	Very satisfied	55 (38.5)
	Somewhat satisfied	51 (35.6)
	Neither satisfied nor dissatisfied	22 (15.4)
	Somewhat dissatisfied	10 (7.0)
	Very dissatisfied	5 (3.5)

**Figure 3:**
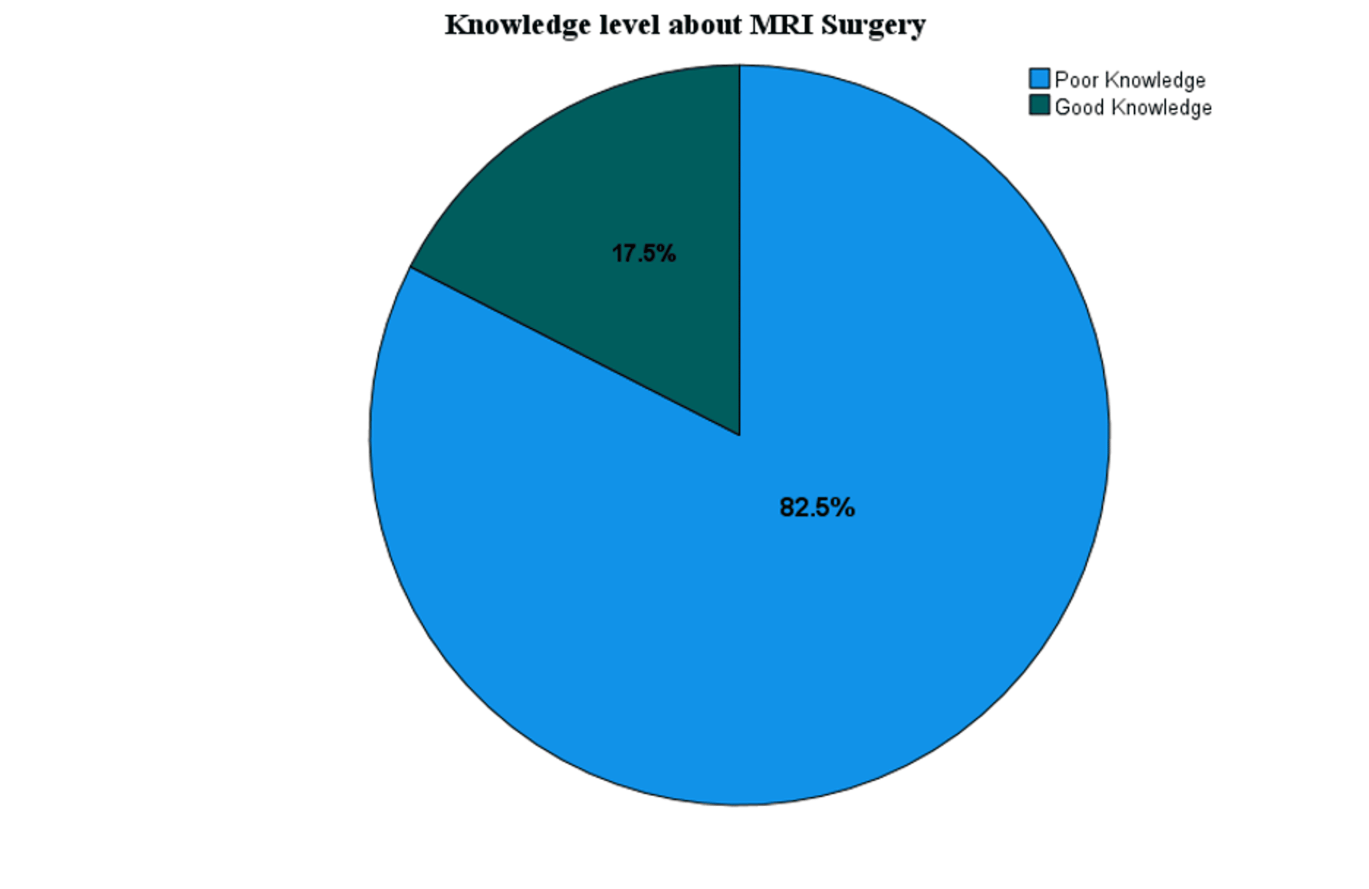
Distribution of knowledge level about MRI scan.

Figure 3 illustrates the distribution of participants' knowledge levels regarding MRI scans. Of the participants, 72 (17.5%) demonstrated good knowledge, while the majority, 340 (82.5%), had poor knowledge.

Table 6 shows the association between socio-demographic factors and knowledge about MRI scans. The study found that a higher proportion of male participants had good knowledge of the MRI scan compared to female participants. Additionally, participants aged 18-25 years demonstrated better knowledge of MRI scans than those in other age groups. Furthermore, individuals with a postgraduate level of education showed a greater understanding of MRI scans than those with lower educational levels. However, no statistically significant differences were observed between these groups regarding knowledge of MRI scan (p-value > 0.05). This suggests that the variations in knowledge are not significantly linked to socio-demographic attributes. It also indicates that the information may not be effectively reaching individuals, underscoring the need for further exploration of other influencing factors and the use of targeted public health campaigns, especially via the internet, to reach a wider population.

**Table 6:** Association between socio-demographic information and knowledge about the MRI scan. The p-value indicates no significant difference between the information groups with respect to their MRI knowledge level, as it exceeds the typical significance threshold of 0.05. Comparison groups include gender, age, education level, and employment status. Chi-square test was utilized; with a p < 0.05 level considered statistically significant. The questionnaire was adapted from Alahmari et al. [13].

Socio-demographic information	Category	Knowledge about the MRI scan		P-value
		Good knowledge, n (%)	Poor knowledge, n (%)	
Gender	Male	24 (21.2)	89 (78.8)	0.216
	Female	48 (16.1)	251 (83.9)	
Age	18-25 years	55 (20.4)	214 (79.6)	0.073
	26-39 years	12 (13.6)	76 (86.4)	
	More than 40 years	5 (9.1)	50 (90.9)	
Education level	Bachelor’s degree	37 (16.0)	194 (84.0)	0.740
	Diploma	6 (16.2)	31 (83.8)	
	High school	22 (19.5)	91 (80.5)	
	Postgraduate	7 (22.6)	24 (77.4)	
Employment status	Employee	15 (14.4)	89 (85.6)	0.149
	Unemployed	9 (12.0)	66 (88.0)	
	Students	48 (20.6)	185 (79.4)	

## Discussion

MRI is a crucial diagnostic tool widely utilized for its non-invasive and detailed imaging capabilities [10]. Studies reveal that despite its vital role in modern medicine, patients’ awareness and knowledge of safety concerning MRI scans are often underexplored [14]. This study evaluates these aspects among patients in the Qassim region, aiming to uncover gaps in understanding and address safety concerns associated with MRI procedures. The study found that slightly more than half the participants, 238 (57.8%), believe they know MRI risks and safety needs. The knowledge levels in this study are higher than those of a survey by Alghamdi, who revealed that only 39.5% of the study participants had adequate knowledge about MRI [14]. The disparity could be attributed to population differences between the two studies.

Over half of the participants, 234 (56.8%), believe that magnetic and radio frequency fields have side effects. This perception indicates a prevalent concern about the potential adverse effects of MRI, which may influence patients' willingness to undergo the procedure. In terms of access to information, the results reveal a significant gap in information about MRI as more than half of the participants, 231 (56.1%), had not received information about MRI risks and safety precautions, perhaps due to inadequate awareness programs and lack of national safety campaign or education materials at imaging centers. For those who had information, the internet emerged as the most frequently cited source (100, 55.2%), followed by media (73, 40.3%). This suggests that digital platforms are crucial in disseminating MRI safety information, which aligns with broader trends observed in other studies where digital sources and direct communication are preferred for health information dissemination [15,16].

Our results reveal that only one-third of the participants, 143 (34.7%), had undergone an MRI examination before, with the majority of those who had undergone the process having done it once (85, 59.4%). The actual experiences regarding MRI safety and precautions were positive, with nearly two-thirds (93, 65.0%) of the participants asserting their experience matched their expectations and significantly increased their understanding of MRI safety and risks. Interestingly, nearly three-quarters (106, 74.1%) of the participants were satisfied with the MRI safety, with only a negligible proportion (5, 3.5%) expressing dissatisfaction. Consistent with our study, a study conducted in Najran asserted that MRI is a safe method of evaluation and is superior to other tests in terms of safety and dependability [3].

It was evident that social demographic factors did not influence knowledge about MRI surgery; none of the variables - gender, age, education level, or employment status - demonstrated statistical significance, as all p-values were above the conventional threshold of 0.05. For gender, males showed slightly better knowledge (24, 21.2%), compared to females (48, 16.1%), but this was not statistically significant (p = 0.216). Similarly, younger individuals aged 18-25 years had better knowledge (55, 20.4%) than older age groups, yet this difference was not statistically significant (p = 0.073). Contrary to our findings, Farinha et al. found that being female and having a higher education level were significant predictors of knowledge about MRI [17]. The disparity could be caused by various contextual and cultural differences between the study populations. The sample in the Qassim region may have different levels of access to information or health education resources compared to the population studied by Farinha et al. Additionally, another explanation for the absence of significant associations is the relatively homogeneous nature of our sample, which was predominantly young and highly educated. Such limited variability may have reduced the ability to detect meaningful differences between demographic groups.

The primary limitation of the study was its cross-sectional design, which allows for the identification of associations between variables but does not establish causality. The utilization of online questionnaires, which tend to exclude older or less technologically literate participants, is likely to introduce coverage bias, resulting in skewed results. Additionally, the use of these online surveys requires providing accurate responses without the opportunity to verify their answers, potentially introducing bias.

## Conclusions

The study concludes that although more than half of the participants believed they were informed about the risks and safety measures associated with MRI, significant gaps in knowledge and information dissemination remain. Interestingly, the majority of participants who had undergone an MRI reported that their actual experiences met their expectations and increased their understanding of MRI safety. The social demographic factors, such as gender, age, education level, and employment status, did not significantly influence their knowledge levels. These findings highlight the need for targeted educational interventions to ensure all patients, regardless of their background, are well-informed about MRI procedures. Given that the internet is a major source of information among the population, leveraging online platforms and social media could effectively reach a broader audience.

## References

[1] Alelyani M, Gameraddin M, Alasmari A, Alshahrani F, Alqahtani N, Musa A (2021). Patients' perceptions and attitude towards MRI safety in Asir Region, Saudi Arabia. Patient Prefer Adherence.

[2] Wally SF, Alwabisi SAO, Aljohani LMM (2023). Assessment of the knowledge and attitude regarding magnetic resonance imaging safety among the general population in Saudi Arabia. Med Sci.

[3] Asiri AAM (2022). Awareness and knowledge of MRI safety among radiological students, interns, fresh graduates and trainees. Russ Open Med J.

[4] Alelyani M, Alqahtani M, Alamri S (2021). Saudi Arabian health workers’ perception and attitudes toward magnetic resonance imaging safety. J Radiol Nurs.

[5] Alhazmi FH, Alsharif WM, Alrehily FA (2023). Prospective health professionals’ knowledge, awareness, attitude, and practice concerning MRI safety. Open Public Health J.

[6] Kim SJ, Kim KA (2017). Safety issues and updates under MR environments. Eur J Radiol.

[7] Shrestha S, Khadka B (2020). Assessment of patients’ knowledge, perception and safety regarding MRI scan. J Manmohan Mem Inst Health Sci.

[8] Hussin IP, Hussin MC, Jaha SS (2023). Investigating the MRI safety knowledge of healthcare workers: a cross-sectional study. Int J Med Phar Drug Res.

[9] Al-Radaideh A, Al-Modallal H (2023). MRI safety matters: assessing the knowledge of radiologic technologists and nurses for safe imaging practices. J Radiol Nurs.

[10] Alyami A, Hakami MM, AboTalib MM (2023). Evaluation of awareness and knowledge regarding MRI safety among students in the faculty of applied medical science at Jazan University. J Radiat Res Appl Sci.

[11] Abd Aziz NN, Zakaria WFWC, Yamin LSM (2022). Assessment of knowledge and perception toward magnetic resonance imaging (MRI) safety among healthcare workers. Healthscope.

[12] Munn Z, Jordan Z (2011). The patient experience of high technology medical imaging: a systematic review of the qualitative evidence. JBI Libr Syst Rev.

[13] Alahmari DM, Alsahli FM, Alghamdi SA, Alomair OI, Alghamdi A, Alsaadi MJ (2022). Assessment of patient knowledge level towards MRI safety before the scanning in Saudi Arabia. Int J Gen Med.

[14] Alghamdi SA (2024). Assessment of patients' knowledge and perceptions of MRI scans and safety in Saudi Arabia. Front Public Health.

[15] Heye T, Knoerl R, Wehrle T (2020). The energy consumption of radiology: energy- and cost-saving opportunities for CT and MRI operation. Radiology.

[16] Stoumpos AI, Kitsios F, Talias MA (2023). Digital transformation in healthcare: technology acceptance and its applications. Int J Environ Res Public Health.

[17] Farinha MN, Semedo CS, Diniz AM, Herédia V (2023). Individual and contextual variables as predictors of MRI-related perceived anxiety. Behav Sci (Basel).

